# Prevalence and risk factors of intestinal protozoan infections: a population-based study in rural areas of Boyer-Ahmad district, Southwestern Iran

**DOI:** 10.1186/s12879-016-2047-4

**Published:** 2016-11-25

**Authors:** Bahador Sarkari, Ghasem Hosseini, Mohammad Hossein Motazedian, Mohammad Fararouei, Abdolali Moshfe

**Affiliations:** 1 Basic Sciences in Infectious Diseases Research Center, Shiraz University of Medical Sciences, Shiraz, Iran; 2 Department of Parasitology and Mycology, School of Medicine, Shiraz University of Medical Sciences, Shiraz, Iran; 3 Department of Epidemiology, School of Health, Shiraz University of Medical Sciences, Shiraz, Iran; 4 Cellular and Molecular Research Center, Yasuj University of Medical Sciences, Yasuj, Iran

**Keywords:** Prevalence, Protozoan infections, Southwestern Iran

## Abstract

**Background:**

Parasitic infections are still a significant health problem in rural areas in developing countries including Iran. There is no recent population-based data about the prevalence of human intestinal parasites in most rural areas of Iran. The current study aimed to determine the prevalence of intestinal protozoan infection in inhabitants of rural areas of Boyer-Ahmad district, Southwestern Iran.

**Methods:**

A total of 1025 stool samples were collected from the inhabitant of 50 randomly selected villages in Boyer-Ahmad Township. The stool samples were evaluated by parasitological methods including, direct wet-mounting, formalin ethyl acetate concentration, zinc sulfate floatation, and Trichrome permanent stain for detection of protozoan infections. Diarrheic samples were further evaluated with a modified Ziehl–Neelsen staining method for detection of coccidian parasites.

**Results:**

The prevalence of both pathogenic and nonpathogenic intestinal parasites in the population was 37.5% (385 out of 1025 cases), some individual with multiple infections. *Giardia lamblia* was detected in 179 (17.46%), *Blastocystis hominis* in 182 (17.76%), *Entamoeba histolytica*/*dispar* in 9 (0.87%), *Endolimax nana* in 216 (21.07%), *Entamoeba coli* in 151 (14.73%), *Ioedamoeba butschlii* in 45 (4.39%), *Chillomastix mesnili* in 22 (2.14%), *Trichomonas hominis* in 2 (0.19%) and *Dientamoeba fragillis* in 2 (0.19%) of cases. Multivariate logistic regression revealed significant associations between protozoan infection (pathogenic protozoa) and contact with animals (OR yes/no = 2.22, *p <* 0.001) and educational status (OR higher/illiterate = 0.40, *P =* 0.01).

**Conclusion:**

Findings of this study demonstrated that protozoan infection rate in rural areas of southwestern Iran is still high and remained as a challenging health problem in these areas.

## Background

Helminthes and protozoan infections are still the most common infections worldwide. These infections may cause anemia, malnutrition and other physical and mental impairments in infected people, particularly in children [1]. Intestinal protozoan infections remain as a major health problem in tropical and subtropical areas of the world, especially in rural areas. According to WHO reports there are 450 million people infected with intestinal parasites in the world [1]. Little health awareness, inadequate sanitation and insufficient water supply attributed to high prevalence of intestinal parasites in rural areas in developing countries [1]. Determination of intestinal protozoan infection is essential for the effective implementation of prevention and control programs in combating these neglected protozoan infections. Furthermore, differences in socio-demographic status of people in different communities necessitate the population-based surveys of intestinal parasites in different geographical areas of a given country.

The human intestinal protozoa include nonpathogenic and pathogenic parasites. While pathogenic parasites may cause overt disease in infected individuals, nonpathogenic parasites are still important since their existence point to a fecal-oral transmission in infected people. Moreover, contamination with these nonpathogenic parasites are an index for sanitary and health conditions of individuals in a given area.

The prevalence rates of protozoan infections are quite high in some of developing countries, including Iran [2–7]. Previous studies in Iran revealed high prevalence rate of parasitic infection in different part of the country [8–10]. A large scale survey by Sayyari et al., on 53,955 stool samples from different provinces of the country showed that 19.3% of the study populations are infected with at least one of intestinal parasitic infection [2].

Most of studies regarding the prevalence of parasitic infection in Iran have been done in urban areas, and on defined groups, mainly school age children. Therefore, there is a lack of population-based study to provide information on protozoan infections and factors associated with their transmission and prevalence in most of rural areas or in deprived communities in the country. Lack of such information justified the current study which aimed to assess the prevalence of protozoan infections in rural areas of Boyer-Ahmad district, southwestern Iran, and to investigate the risk factors contributing to the prevalence of intestinal protozoan infections in this area. This area has recently been considered as a new focus of human fascioliasis and most of population-based studies are focusing on this newly emerged parasitic infection, neglecting the other parasitic diseases [11, 12].

## Methods

This cross-sectional study was conducted in 2014 (June to December), in rural areas of Kohgiluyeh and Boyer-Ahmad province, Southwest Iran. The province has a moderate and cool climate. Mean annual rainfall and temperature are 600 mm and 16 °C, with plenty of snow during the cold winter. Wild pistachio, tulips and oak forests covered the ground in the province. Socio-economic conditions make the people to depend more on animal husbandry and farming for their living. Large pastures for animals provide appropriate conditions for transmission and establishment of zoonotic diseases in the area [11, 13–15].

District population composed of 240,000 residents (about 60% in rural and 40% in urban areas), in 50,000 households. Health facilities include governmental primary health care centers.

### Sample collection

A total of 1025 stool samples were collected from the inhabitant of 50 randomly selected villages (with total population of 84816 inhabitants) in Boyer-Ahmad district. Distance of some of these villages to Yasuj, capital of the province, is 150 km. Subjects were called to participate voluntarily in the study after a clear explanation of the objectives of the study was provided by an expert, a MS student who was familiar with language and cultural affairs of the inhabitants of the area. Informed consent was obtained from the participants or their parents, in term of children. The study was approved by Ethics Committee of the Shiraz University of Medical Sciences. Confidentiality of the details of the participants was assured. Demographic information including age, gender, level of education, residence, occupation, health practices, source of water supply, contact with animals, symptoms, and also data related to epidemiology of protozoan infections were collected through a predesigned questionnaire. Stool samples were collected in mouthed screw-capped container. A container was given to each participant. Next morning the containers were collected and transferred to Department of Parasitology at Yasuj University of Medical Sciences. Preliminary detection of protozoa cysts or trophozoite was through direct smear, prepared from the fresh specimens.

Moreover, the stool samples were evaluated by formol-ethyl acetate sedimentation and zinc sulfate floatation techniques. Trichrome permanent stain was also used for detection of protozoan infections. Diarrheic samples were further evaluated with a modified Ziehl–Neelsen staining method for detection of coccidian parasites.

### Statistical analysis

Data were entered into SPSS package for Windows version 20 (SPSS Inc. Chicago. LL, USA). Relationships between variables were examined by chi-square test. Logistic regression analysis was employed to determine the independent risk factor for protozoan infection in the subjects. The level of significance was set at 5%.

## Results

Mean age of participants was 20.25 (±15.86) with age range of 1-89 year. Age group of 1–10 years included most of the subjects (38.63%). From 1025 participants, 473 (46.1%) were male and 552 cases (53.9%) were female.

The overall prevalence of any enteric protozoan in the population was 37.6% (385 out of 1025 cases), some individual with multiple infections. *Giardia lamblia* was detected in 179 (17.46%), *Blastocystis hominis* in 182 (17.76%), *Entamoeba histolytica/dispar* in 9 (0.87%), and *Dientamoeba fragillis* in 2 (0.19%) of cases. Moreover, nonpathogenic protozoan including *Entamoeba coli* was detected in 151 (14.73%), *Ioedamoeba butschlii* in 45 (4.39%), *Endolimax nana* in 216 (21.07%), *Chillomastix mesnili* in 22 (2.14%), and *Trichomonas hominis* in 2 (0.19%) of participants.

Multivariate logistic regression revealed significant associations between protozoan infection (only pathogenic protozoa) and contact with animals (OR yes/no = 2.22, *p <* 0.001) and also educational status (OR higher/illiterate = 0.40, *P =* 0.01). Moreover, using multivariate logistic regression the results showed significant associations between protozoan infection (all types) and contact with animals (OR yes/no = 2.43, *p <* 0.001) age (OR = 0.99, *p =* 0.033) and educational status (OR higher/illiterate = 0.38, *P =* 0.003).

*Giardia lamblia* was the predominant pathogenic protozoa infection (17.46%) among the participants. Univariate analysis was also performed for infection with *Giardia*. Result showed a significant association between *Giardia* infection and age (more prevalent in 1–10 years old subjects), sex (more prevalent in males), educational status (more prevalent in uneducated subjects), and having contact with animals (more prevalent in those who kept animals in their house). Tables 1 and 2 show the prevalence of each protozoa in villagers based on sex and age.

**Table 1:** Prevalence of intestinal protozoan in rural areas of Boyer-Ahmad District, Southwestern Iran, based on sex

Protozoan species	Male No. (%)	Female No. (%)	Total No. (%)	*P-* value
*Giardia lamblia*	96 (20.3%)	83 (15%)	179 (17.46%)	*<0.05*
*Endolimax nana*	88(18.6%)	128 (23.18%)	216 (21.07%)	*<0.05*
*Blastocystis hominis*	78 (16.42%)	104 (18.84%)	182 (17.76%)	*>0.50*
*Entamoeba coli*	60 (12.68%)	91 (16.48%)	151 (14.73%)	*<0.05*
*Iodamoeba butschlii*	20 (4.22%)	25 (4.52%)	45 (4.39%)	*>0.50*
*Chilomastix mesnili*	10 (2.11%)	12 (2.17%)	22 (2.14%)	*>0.50*
*Entamoeba histolytica/dispar*	2 (0.42%)	7 (1.26%)	9 (0.87%)	*>0.50*
*Dientamoeba fragilis*	0 (0.0%)	2 (0.42%)	2 (0.19%)	*>0.50*
*Trichomonas hominis*	1 (0.21%)	1 (0.18%)	2 (0.19%)	*>0.50*

**Table 2:** Prevalence of intestinal protozoan in rural areas of Boyer-Ahmad District, Southwestern Iran, in different age group

Protozoan species	1–10 No. (%)	11–20 No. (%)	21–30 No.(%)	31–40 No. (%)	41–50 No. (%)	>50 No. (%)	Total No. (%)
*Giardia lamblia*	105	27	19	17	4	7	179
	(26.51%)	(13.77%)	(12.02%)	(10.24%)	(6.45%)	(14.89%)	(17.46%)
*Endolimax nana*	72	37	38	38	15	16	216
	(18.18%)	(18.87%)	(24.05%)	(22.89%)	(24.19%)	(34.04%)	(21.07%)
*Blastocystis hominis*	65	29	33	35	11	9	182
	(14.61%)	(14.79%)	(20.88%)	(21.08%)	(17.74%)	(19.14%)	(17.76%)
*Entamoeba coli*	44	31	32	24	9	11	151
	(11.11%)	(15.81%)	(20.25%)	(14.45%)	(11.51%)	(23.40%)	(14.73%)
*Iodamoeba butschlii*	13	5	10	9	2	6	45
	(3.28%)	(2.55%)	(6.30%)	(5.42%)	(3.22%)	(12.76%)	(4.39%)
*Chilomastix mesnili*	5	5	3	6	2	1	22
	(1.26%)	(2.55%)	(1.89%)	(3.61%)	(3.22%)	(2.12%)	(2.14%)
*Entamoeba histolytica/dispar*	2	4	3	0	0	0	9
	(0.5%)	(2.04%)	(1.89%)	(0.0%)	(0.0%)	(0.0%)	(0.87%)
*Dientamoeba fragilis*	0	0	1	0	1	0	2
	(0.0%)	(0.0%)	(0.63%)	(0.0%)	(1.61%)	(0.0%)	(0.19%)
*Trichomonas hominis*	0	0	0	2	0	0	2
	(0.0%)	(0.0%)	(0.0%)	(1.20%)	(0.0%)	(0.0%)	(0.19%)

## Discussion

The current study documented the relatively high prevalence rate of intestinal parasites in inhabitants of rural areas in southwestern Iran. Findings of the study showed that more than 30% of the inhabitants of rural areas in Boyer-Ahmad district in southwest of Iran are infected with at least one of intestinal parasites, although some of them were non-pathogenic. This finding is consistent with other studies performed in different geographical areas of Iran [2, 9, 16, 17]. In Iran, results of previous studies indicated that *Giardia* and *Blastocystis* are the most common intestinal protozoan infection among the population [2, 8–10, 16, 17]. The most prevalent intestinal parasite in the current study was *Giardia* and *Blastocystis* which were rated at 17.4 and 17.7% respectively. Study of Pestechian et al., in Isfahan central Iran, revealed a prevalence rate of 28% for *Giardia* and 27.5% for *Blastocystis* infections [18]. A study on 1041 stool samples of food handlers in northern Iran revealed *Giardia* in 53.9 and *Blastocystis* in 13% of cases [19].

A study on 1100 school age children in Sari, northern Iran, documented a prevalence rate of 10.6 for *Giardia* and 13.5% for *Blastocystis* among the children [9]. In a comprehensive study in Karaj City, next to Tehran, stool samples of 13915 inhabitant of the city were assessed for intestinal protozoan infection. *Giardia* was the most predominant infection (3.8%) found in the stool samples of recruited subjects [16].

Results of the current study indicate that intestinal parasitic infections are still a significant public health problem in Iran, same as other developing countries. In a study in Riyadh, Saudi Arabia, the prevalence rate of intestinal parasitic infection was found to be 32.2% in all population and 34.4% among children under 12 years old [20]. The prevalence rates of parasitic diseases in some of other developing countries are higher than those reported in the current study [3, 5, 21]. Study on 200 children, aged 5–15 years in Cuba, revealed that 38.5% of the children are infected by *Giardia*. The study also showed that 93.8% of children in rural areas are infected with at least one of the parasitic infection [22].

All of intestinal parasites reported in our study have fecal-oral transmission route. *Giardia* and *Blastocystis* which were the most frequent infection in this study; can be transmitted via the fecal-oral route. Contaminated water supply, soil or vegetables might be the main sources of infection for the villagers in this study.

Although still high, but rate of intestinal protozoa infections in the current study decreased in comparison with the other studies conducted in the region or in other regions of the country with relatively similar conditions [10]. This might be explained by the fact that the rural areas of the district had experienced economic and social changes during the last few decades, resulted in a marked improvement in sanitary situation and health status of the inhabitants, even though level of sanitation is still rudimentary.

Rates of infection with intestinal parasites were also seen to decrease during the last decades in other parts of Iran. Recent study on children referred to medical centers in Tehran showed that the rate of intestinal protozoan infections reduced from 14.9% to 4.3% in 2000–2008 compared to 1991–2000 [8].

In this study a significant association was found between intestinal parasites and job, educational status of people and also having contact with animals. Significant association between people educational status and rate of other infectious diseases has also been reported in this area [23–25]. Educated people are more aware of transmission of protozoan infection and they may apply the necessary measurements to avoid the infection. Increase of health awareness among the community is crucial and is an important requirement for prevention and control of parasitic diseases including intestinal protozoan in this and any other similar areas of the country [26]. A case control study in south of Tehran revealed that knowledge and practices of children significantly reduced the rate of infection in group covered by health education program [27]. In Yaman and Nepal, the practice of hand washing had a strong association with the prevalence of protozoan parasites [3, 21]. In Iran, the country has been encountered by deceased level of rainfall during the last decades and water shortages are expected to remain a key resource problem in the country. It is obvious that scarcity of water results in poor sanitation and affects the people’s health.

Regarding the people occupation, infections were more prevalent in farmers and those who practice animal husbandry. This is not surprising since these people have more exposure to the source of infections which are usually soil and vegetables. Rate of pathogenic protozoan in remote area were higher. This is linked to the health facilities and also sanitary conditions which are usually low in such areas.

## Conclusion

Findings of the current study showed high prevalence of intestinal protozoa infection in residents of rural areas in southwest of Iran, with *Giardia* and *Blastocystis* being the most prevalent infections among the villagers. Contaminated soil and vegetables along with inadequate information about risk factors of transmission seem to play major roles in the transmission of intestinal protozoan among the villagers. These factors must be considered for proper implementation of any prevention and control strategies regarding the intestinal protozoan in these and any other areas with similar condition.
